# Investigation of the Lactic Acid Bacteria in Kazak Cheese and Their Contributions to Cheese Fermentation

**DOI:** 10.3389/fmicb.2020.00228

**Published:** 2020-03-12

**Authors:** Jie Li, Qian Huang, Xiaochun Zheng, Zhengkai Ge, Ke Lin, Dandan Zhang, Yu Chen, Bin Wang, Xuewei Shi

**Affiliations:** Food College, Shihezi University, Shihezi, China

**Keywords:** Kazak cheese, lactic acid bacteria, enzyme, physical and chemical indicators, aroma

## Abstract

Kazak cheese is a traditional dairy product fermented by lactic acid bacteria (LAB) in Xinjiang. To investigate the LAB in Kazak cheese and their contributions to cheese fermentation, four representative LAB, *Streptococcus thermophilus* B8, *Lactobacillus helveticus* B6, *Weissella confusa* B14, and *Lactobacillus rhamnosus* B10, were isolated from Kazak cheese and subsequently used to ferment cheeses, which were named StC, LhC, WcC, and LrC, respectively. The result showed that most of the physical and chemical indicators had no significant difference, except for moisture and fat. *W. confusa* B14 was beneficial to the production of amino acids, whereas *S. thermophilus* B8 promoted the formation of organic acids and contributed to formation ideal texture property. Furthermore, the four cheeses all possessed a strong fruity aroma, with brandy, sweet, herbaceous, pungent, and fatty aromas being the most prominent in WcC. This is because *L. helveticus* B6 produced a high concentration of hexanal, nonanal, octanal, 3-methylbutanoic acid, ethyl acetate, ethyl butanoate, isoamyl acetate, and ethyl hexanoate in LhC. Research on the fermentation mechanism of LAB in cheese will provide a theoretical basis for the quality control and industrial production of Kazak cheese.

## Introduction

Kazak cheese, a hard cheese handmade from milk or goat milk, is a traditional fermented dairy product of the Kazak in Xinjiang, China. Because of a relatively lower standard of living, Kazak cheese not only extends the consumption of dairy products but also serves as an excellent carrier for various probiotics. Like Kopanisti cheese in Greece (Rozos et al., 2018) and Kurdish cheese in Khorasan (Milani et al., 2017), Kazak cheese is spontaneously fermented by naturally occurring lactic acid bacteria (LAB) without the addition of a starter culture.

Lactic acid bacteria, which colonize animals during the suckling period, are important bacteria in raw animal milk. The microflora in raw camel milk consists of *Streptococcus*, *Lactococcus*, *Weissella*, *Pediococcus*, *Lactobacillus*, and *Enterococcus* (Fguiri et al., 2016; Fugl et al., 2017). However, yak milk contains *Lactobacillus* species, *Enterococcus* species, *Lactococcus* species, *Leuconostoc* species, and *Weissella cibaria* (Kaur et al., 2017), whereas *Lactococcus*, *Lactobacillus*, *Leuconostoc*, *Streptococcus*, *Enterococcus*, *Macrococcus*, *Weissella*, and *Bacteroidetes* were the dominant flora in raw milk (Gao et al., 2017). Furthermore, LABs function as a starter culture in raw animal milk and contribute to the fermentation of cheese, kefir, and yogurt. Zheng et al. (2018b) observed that *Streptococcus* and *Lactobacillus* species were the primary strains in Kazak cheese. In buffalo mozzarella cheese, the main LABs were identified as *Streptococcus thermophilus*, *Lactobacillus fermentum*, *Enterococcus faecium*, *Leuconostoc mesenteroides*, *Lactobacillus casei*, *Lactobacillus delbrueckii*, *Enterococcus durans*, and *Lactobacillus helveticus* (Silva et al., 2015). In traditional yogurt, LAB strains consist of *S. thermophilus, L. delbrueckii, L. mesenteroides*, and *Lactobacillus plantarum* (İspirli and Dertli, 2018), whereas *Lactobacillus*, *Lactococcus*, and *Streptococcus* species are widespread in milk kefir (Rosa et al., 2017).

Lactic acid bacteria can be used as a starter culture for cheese fermentation because of their ability to release proteases, lipases, or β-galactosidases to form a unique taste, aroma, and texture (Juan et al., 2016). During cheese fermentation, the most important features and functions of LABs are (1) the conversion of lactose in milk into small molecular monosaccharides, galactose, and glucose, which promote the formation of cheese flavor (Blaya et al., 2018); (2) the degradation of proteins into peptides and free amino acids (FAAs) in cheese; and (3) the decomposition of lipids into fatty acids (Konkit and Kim, 2016). Proteases are key enzymes in the hydrolysis of protein during cheese fermentation. Importantly, aspartic protease is a special common chymosin that digests κ-casein to promote milk coagulation, thereby improving the taste, flavor, and functional properties of cheese (Ozturkoglu-Budak et al., 2016). Lipases are capable of gradually hydrolyzing triglycerides into glycerol and fatty acids (Mohamed et al., 2011). β-Galactosidase, also known as lactase, is responsible for the hydrolysis of lactose to glucose and galactose. Ozturkoglu-Budak et al. (2016) indicated that proteases, lipases, or β-galactosidases in LABs contribute to the formation of cheese flavors by releasing fixed volatile compounds, producing a distinctive odor, or modifying the primary aroma. For example, methyl ketones, aldehydes, ethyl esters, alcohols, sulfur compounds, carboxylic acids, and aromatic hydrocarbons were the typical aroma of cheese, which were produced by proteolytic and lipolytic pathways and by the metabolism of lactate and citrate (Bertuzzi et al., 2018). During cheese fermentation, lactic acid and acetic acid significantly increase with bacterial adjuncts compared with *Lactococcus*; however, other acids are not influenced by bacterial adjuncts (Murtaza et al., 2017).

Kazak cheese is a unique traditional fermented food in Xinjiang, China, and is rich in a variety of microorganisms. To investigate the effects of LABs on the physical and chemical properties and flavor of Kazak cheese, *Lactobacillus rhamnosus* B10, *S. thermophilus* B8, *L. helveticus* B6, and *Weissella confusa* B14 were isolated from Kazak cheese and were used for cheese fermentation individually, to explore the influence of LABs on cheese quality and flavor and provide theoretical basis for the quality control and industrial production of Kazak cheese.

## Materials and Methods

### Isolation of LABs

Cheese samples ripened for a period of at least 3 mon were collected from Altay in Xinjiang (Hazak manual cheese), packaged, labeled, and placed in a car refrigerator at −4°C, transported back to the laboratory within 18 h, and stored at 4°C. LAB in Kazak cheese were screened on MRS (peptone 10 g/L, meat extract 8 g/L, yeast extract 4 g/L, glucose 20 g/L, sodium acetate 5 g/L, triammonium citrate 2 g/L, tween 80 1 g/L, magnesium sulfate 0.58 g/L, manganese sulfate 0.28 g/L, and agar 10 g/L) medium for 48–72 h at 37°C (Plessas et al., 2017). All isolates were further purified by streaking and ultimately identified by their morphological and staining characteristics. All LABs were stored at −20°C in tubes containing 30% sterile glycerol.

### Identification of LABs

Genomic DNA was extracted using a DNA extraction kit (TransGen Biotech, Beijing, China) according to the manufacturer’s instructions. The primers 27F (5′-AGAGTTTGA TCCTGGCTCAG-3′) and 1492R (5′-TACGGCTACCTTGTTA CGACTT-3′) (Sangon Biotech, Shanghai, China) were used for the amplification of 16S rDNA. The total volume of each PCR system was 50 μL, consisting of 25 μL 2 × Taq enzyme, 1.0 μL primer 27F (10 mmol/L), 1.0 μL primer 1492R (10 mmol/L), 2 μL DNA template (100 ng/μL), and 21 μL dd H_2_O. The amplification was performed in a PCR Thermocycler (D-37085, Göttingen, Germany) using the following program: 94°C for 4 min, followed by 30 cycles of denaturation at 94°C for 45 s, annealing at 55°C for 40 s, and extension at 72°C for 30 s. Finally, a final extension cycle was performed at 72°C for 10 min. Agarose gel (1%) electrophoresis was used to confirm the amplification effect and fragment size. The PCR products were purified and sent to Shanghai Shenggong Biotechnology Co., Ltd., for sequencing. Nucleotide sequences were analyzed with the BLAST search tool in the GenBank database for similarity comparisons^1^.

### Screening LABs in Kazak Cheese With Enzyme Activity

Protease-producing LABs were screened by adding 2% skim milk powder (Yuanye Biotechnology Co., Ltd., Shanghai, China) to MRS agar plates; after cultivation, the formation of a white transparent halo around the colony indicated a positive protease-producing strain (Ozturkoglu-Budak et al., 2016). Protease activity was evaluated according to Kim’s method (Kim et al., 2013).

The qualitative evaluation of lipase activity of all strains was determined on MRS agar plates prepared with 10% tributyrin (Yuanye Biotechnology Co., Ltd.), and the formation of a transparent halo on the plate indicated that the microorganisms metabolized the lipids. Lipase activity was measured by potentiometric titration with an indicator (Konkit and Kim, 2016).

Strains were screened for β-galactosidase activity by adding 20 mg/mL 5-bromo-4-chloro-3-indolyl β-D-galactopyranoside (X-gal) (Yuanye Biotechnology Co., Ltd.) to MRS plates, and positive strains were selected by the formation of a blue colony after incubation. Quantitative assays of β-galactosidase activity were performed according to Xiaoji Zheng’s method (Zheng et al., 2018a).

### Cheese Making and Sampling

Twenty liters of standardized milk (3.5% fat) (Garden Dairy Co., Ltd., Xinjiang, China) was pasteurized at 65°C for 30 min with constant stirring. After being cooled to 35°C, the milk was divided into four parts with 5 L in each. Four trials of cheese making were conducted separately using four different lactic acid starters (*S. thermophilus* B8, *L. rhamnosus* B10, *W. confusa* B14, and *L. helveticus* B6). All of the strains were activated with milk at 35°C and added to the milk at a cell concentration of approximately 1.0 × 10^7^ colony-forming units (cfu)/mL. The starting cultures were individually added to the milk, and then 0.02% chymosin (Hebei Gebeida Biological Technology Co., Ltd., Hebei Province, China) was added to the milk to induce curd formation with all fermentation processes performed under sealed conditions. Then, the curd block was cut into a 1-cm square to discharge the whey, placed in four layers of gauze to filter out the whey, and placed into a mold to further remove the whey by pressure. After the whey was removed, the curd was immersed in 2% (wt/vol) saline for 1 h and then dried at room temperature for 48 h. The cheese was collected and stored at 4°C.

Samples were obtained periodically at intervals of 0, 8, 16, 24, and 32 days. Then, the samples were placed in a 50-mL centrifuge tube, frozen in liquid nitrogen, and finally stored in a −80°C refrigerator until analysis.

### Determination of the Physical and Chemical Indicators

The protein content was determined by the Kjeldahl method (McCarthy et al., 2017) with a Kjeldahl instrument (KjeIMaster K-375; Switzerland BÜCHI Laborechnik AG, Switzerland). Moisture measurements were carried out by the drying method. The fat content was measured by Soxhlet extraction. Silver nitrate precipitation titration was used to determine the salt content. The pH of the cheese was evaluated with a calibrated electronic digital pH meter (PHS-3C; Shanghai Jingke Co., Ltd., Shanghai, China). Acidity was determined by indicator titration according to GB/T 5009.239-2016, and experiments were repeated in triplicate throughout the study.

### Determination of Amino Acids

An amino acid analyzer (LBA800; Rambo Co., Ltd., United States) was used to determine the FAA content according to the Chinese standard method GB/T 5009.124-2016. The amino acids were detected at wavelengths of 440 nm (proline and hydroxyproline) and 570 nm (all other FAAs). The quantification of amino acids was performed based on comparing the peak area observed for a standard mixture with a known concentration (Niro et al., 2017).

### Determination of Organic Acids

Organic acids were quantified by high-performance liquid chromatography (LC-2010; Shimadzu, Japan) equipped with a Spursil C18 (LC) column (250 mm × 4.6 mm × 5 μm) (Dima Technology Co., Ltd., Guangzhou, China). The mobile phase was 0.1% phosphoric acid–methanol. The flow rate of methanol was 0.2 mL/min for 10 min, 0.3 mL/min for 5 min, and finally increased to 0.4 mL/min for 10 min. The injection volume was 20 μL, the UV detection wavelength was 210 nm, and the column temperature was 40°C (Murtaza et al., 2017). Organic acids were assessed with external standard curves of tartaric acid, lactic acid, malic acid, citric acid, succinic acid, propionic acid, and pyruvic acid (Shanghai Yuanye Biotechnology Co., Ltd.).

### Determination of Texture

Texture analysis was executed with a TA texture analyzer (TA. Xtplus; Micro Stable System Co., Godalming, United Kingdom) and a P/36R cylindrical probe (TA15/1000, 458 Å, 36-mm diameter). Cheese samples were removed from the refrigerator at 4°C and equilibrated for 30 min at room temperature before measurement (Rehman et al., 2018). Then, cheeses were cut into 2 × 0.5-cm cubes and placed vertically in the middle of the container. Measurement conditions were as follows: test mode: TPA; pretest speed: 5 mm/s; test speed: 1 mm/s; speed after test: 5 mm/s; trigger force: 5 g; interval between two presses: 5 s; and target mode: deformation 50% (Bekele et al., 2019). The test required two consecutive presses with a test speed of 1 mm/s.

### Determination of Volatile Compounds

Solid-phase microextraction gas chromatography–mass spectrometry (SPME/GC-MS; American Agilent Co., Ltd., Palo Alto, CA, United States) was used to extract and detect volatile compounds in the cheese (Tian et al., 2017; Mugampoza et al., 2019).

Each cheese sample (1.5 g) was uniformly mixed with 0.2 g of sodium chloride and 0.2 g of anhydrous sodium sulfate and before being transferred to an extraction vial, with 1 μL 2-octanol (40 μg/kg) subsequently added as an internal standard. Then, 1-cm Stable flex 50/30 μm SPME fiber (DVB/CAR/PDMS 50/30 μm; Supelco, Bellefonte, PA, United States) was inserted into a vial and exposed to the headspace for adsorption for 40 min at 40°C to extract the volatile compounds. Finally, the sample was injected into the GC at an interface temperature of 230°C for 5 min. The volatile compounds were analyzed by an HP INNOWAX 30 m × 0.25-mm capillary column (Agilent Technology Co., Ltd.). The carrier gas was helium at a flow rate of 1 mL/min, and the electron impact was set to 70 eV. The temperature procedure was as follows: 3 min at 50°C, 2°C/min to 100°C, 4°C/min to 180°C, and 10°C/min to 230°C. The identification of the flavor compounds was performed by matching the retention index with those in the National Institute of Standards and Technology mass spectrometry library, and only matches with a match score of 800 or greater were retrieved and recorded. The area normalization method was used to express the content of the volatile components. And the concentration of each compound was obtained by multiplying the internal standard concentration by the ratio of the peak area of each compound to the peak area of the internal standard.

### Statistical Analysis

All data were expressed as the means ± standard deviation of three trials for each sample. Significant differences between the groups were assessed with Duncan multiple range tests and least significant difference tests using IBM SPSS statistics software version 22 (IBM Corp., Armonk, NY, United States). R i386 3.4.2 was used to generate a heatmap to analyze the trend in flavor during the fermentation of the cheese samples. Principal component analysis (PCA) was used to reduce data dimensions using SIMCA 14.1 software (Biometric Software Developer Umetrics, Umeå, Sweden).

## Results

### Screening LABs in Kazak Cheese

A total of 112 bacteria were screened from Altai Kazak cheese by streak plate method. Gram staining showed that 78 strains were Gram-positive, and the identification results showed that the primary strains belonged to *L. plantarum*, *L. rhamnosus*, *L. paracasei*, *L. case*i, *L. helveticus*, *W. confusa*, *Leuconostoc lactis*, *S. thermophilus*, *Lactococcus garvieae*, and *Lactococcus lactis* (Supplementary Table S1). Finally, 18 strains of enzyme-producing LABs were obtained, of which 16 strains produced proteases, eight strains produced β-galactosidases, and only 10 strains produced lipases.

Based on the results of the enzyme activity assay (Supplementary Table S2), *L. rhamnosus* B10, *S. thermophilus* B8, *W. confusa* B14, and *L. helveticus* B6 were selected for further study (Supplementary Figure S1), and their enzyme activities were assessed in greater detail. The activities of protease, lipase, and lactase in *S. thermophilus* were 124, 87, and 162 U/mL, respectively. Both *W. confusa* and *L. helveticus* produced proteases and lipases. *L. rhamnosus* exhibited both protease and lactase activities. Among them, the protease activity of *W. confusa* was as high as 139 U/mL, and the lipase activity of *L. helveticus* was as high as 115 U/mL. Nevertheless, the strain with the highest lactase activity was *L. rhamnosus*, reaching 186 U/mL.

### Physical and Chemical Indicators of the Cheese Fermented by Different Strains

Murtaza et al. (2017) suggested that microorganisms in cheese play a key role in cheese quality. To evaluate the effects of the LABs on cheese quality, *W. confusa* B14, *L. helveticus* B6, *S. thermophilus* B8, and *L. rhamnosus* B10 were independently added into commercially available milk, and cheese was fermented according to the traditional manufacturing process of Kazak cheese in Xinjiang. WcC, LhC, StC, and LrC represented the cheese fermented with *W. confusa* B14, *L. helveticus* B6, *S. thermophilus* B8, and *L. rhamnosus* B10, respectively. Microbiology analysis showed that similar trends in the amounts of LABs were observed in the four cheeses (Supplementary Figure S2). After fermentation, the number of viable bacteria in the fermentation cheese reached approximately 1 × 10^8^ to 1 × 10^9^ cfu/g. During the whole fermentation period from 0 to 32 days of ripening, a slight peak at day 16 occurred, while the numbers decreased by approximately 0.5 log_10_ units from days 16 to 32, which is probably due to self-inhibition by acidification (Supplementary Figure S2). Physical and chemical indicators, including the proteins, moisture, fat, salt, pH, and acidity of the cheese, were measured (Table 1). There was no significant difference in the proteins, salt content, pH, and acidity between the four types of cheese, but the moisture content in WcC was generally higher than that in the other cheeses. Except for moisture and fat, most of the physical and chemical indicators of the cheese produced by different bacteria showed no significant difference.

**TABLE 1:** Physical and chemical indicators of cheese made from different strains.

**Composition**	**LrC**	**StC**	**WcC**	**LhC**
Protein (%)	20.46 ± 1.37^a^	21.74 ± 1.17^a^	22.53 ± 1.53^a^	20.86 ± 0.97^a^
Moisture (%)	16.34 ± 0.34^b^	16.85 ± 0.32^b^	17.58 ± 0.44^a^	16.65 ± 0.41^b^
Fat (%)	29.85 ± 0.63^a^	28.65 + 0.58^b^	30.21 ± 0.79^a^	27.97 ± 0.41^b^
Salt (%)	1.90 ± 0.12^a^	1.90 ± 0.17^a^	1.91 ± 0.12^a^	1.89 ± 0.09^a^
pH	4.98 ± 0.53^a^	4.78 ± 0.49^a^	4.83 ± 0.38^a^	4.85 ± 0.35^a^
Acidity (%)	0.98 ± 0.03^a^	0.97 ± 0.05^a^	1.02 ± 0.06^a^	0.96 ± 0.02^a^

*Data are expressed as the mean ± standard deviation from three replicate analyses (*n* = 3) of three replicate samples. The different lowercase letters in each row indicate a significant difference between the samples (*P* < 0.05).*

### Amino Acids in the Cheese

During the cheese fermentation process, proteases from microorganisms degrade the proteins in milk and produce large amounts of peptides that can be further degraded into small peptides and FAAs (Niro et al., 2017; Moser et al., 2018). The concentration of different FAAs in cheese depends on the milk, starter, rennet, ripening conditions, and time of maturation (Tavaria et al., 2003; Tofalo et al., 2015; McCarthy et al., 2017; Zhou et al., 2018). Furthermore, FAAs are an important component in cheese. To evaluate the contributions of the selected microorganisms to the FAAs, the composition and relative content of FAAs were determined (Table 2).

**TABLE 2:** Free amino acid content in the cheeses (g/100 g).

**FAA**	**LrC**	**StC**	**WcC**	**LhC**
Asp	0.157 ± 0.002^c^	0.163 ± 0.001^b^	0.265 ± 0.002^a^	0.165 ± 0.001^b^
Thr	0.084 ± 0.001^d^	0.095 ± 0.002^b^	0.156 ± 0.001^a^	0.092 ± 0.002^c^
Ser	0.098 ± 0.002^c^	0.112 ± 0.002^b^	0.197 ± 0.002^a^	0.115 ± 0.002^b^
Glu	0.388 ± 0.002^c^	0.392 ± 0.003^c^	0.608 ± 0.004^a^	0.415 ± 0.005^b^
Gly	0.046 ± 0.001^b^	0.049 ± 0.001^b^	0.076 ± 0.002^a^	0.047 ± 0.002^b^
Ala	0.080 ± 0.001^c^	0.086 ± 0.002^b^	0.132 ± 0.003^a^	0.081 ± 0.003^c^
Cys	0.008 ± 0.000^c^	0.013 ± 0.001^b^	0.018 ± 0.001^a^	0.007 ± 0.000^c^
Val	0.147 ± 0.003^c^	0.160 ± 0.003^b^	0.227 ± 0.004^a^	0.144 ± 0.002^c^
Met	0.056 ± 0.002^c^	0.062 ± 0.002^b^	0.098 ± 0.001^a^	0.056 ± 0.001^c^
Ile	0.126 ± 0.003^c^	0.137 ± 0.003^b^	0.197 ± 0.002^a^	0.123 ± 0.002^c^
Leu	0.222 ± 0.004^c^	0.244 ± 0.003^b^	0.261 ± 0.004^a^	0.225 ± 0.005^c^
Tyr	0.101 ± 0.002^d^	0.121 ± 0.001^b^	0.209 ± 0.003^a^	0.105 ± 0.002^c^
Phe	0.112 ± 0.003^c^	0.126 ± 0.003^b^	0.188 ± 0.003^a^	0.113 ± 0.004^c^
Lys	0.184 ± 0.003^c^	0.200 ± 0.003^b^	0.288 ± 0.004^a^	0.183 ± 0.005^c^
His	0.063 ± 0.002^b^	0.068 ± 0.003^b^	0.099 ± 0.005^a^	0.062 ± 0.002^b^
Arg	0.077 ± 0.003^c^	0.087 ± 0.003^b^	0.134 ± 0.001^a^	0.078 ± 0.002^c^
Pro	0.168 ± 0.002^c^	0.186 ± 0.001^b^	0.278 ± 0.002^a^	0.169 ± 0.002^c^
Total	2.117 ± 0.021^d^	2.301 ± 0.032^b^	3.431 ± 0.028^a^	2.180 ± 0.035^c^

*Data are expressed as the mean ± standard deviation from three replicate analyses (*n* = 3) of three replicate samples. The different lowercase letters in each row indicate a significant difference between the samples (*P* < 0.05).*

The contents of Asp, Glu, Val, Ile, Leu, Tyr, Phe, Lys, and Pro in all cheeses reached 0.1 g/100 g, whereas the levels of other FAAs remained low. Among the FAAs in the four types of cheese, the Glu content was the highest, whereas that of Cys was the lowest. This result indicated that Glu was a main FAA in cheese fermented by LABs. Interestingly, all FAAs were higher in WcC than in other cheeses, which may be attributed to its high protease activities. Furthermore, the total FAA (TFAA) content in WcC was the highest (up to 3.43 g/100 g) because of its high protease activity. Moreover, the produced FAAs can contribute to cheese flavor. Glu reacts via the action of γ-glutamyl transferase and provides umami flavor; Val, Met, Ile, Phe, Lys, Leu, Arg, His, and Tyr generate bitterness (Zhao et al., 2016), whereas Ser, Pro, Gly, and Ala provide a sweet taste (Kong et al., 2017). This result suggested that LABs might contribute to cheese flavor by modifying the composition and relative content of amino acids in cheese.

### Organic Acids in the Cheese

During cheese fermentation, most LABs exert an antibacterial effect because of their abilities to produce lactic acid and reduce the pH in the environment (Dalié et al., 2010; Delavenne et al., 2012; Belguesmia et al., 2014). The produced organic acids can be esterified with alcohols, which provide an enhanced aroma and reduce the irritating taste of organic acids (Hasan et al., 2006). Organic acids are the main components in cheese. In this study, the organic acids tartaric acid, malic acid, lactic acid, citric acid, pyruvate acid, propionic acid, and succinic acid were measured (Table 3).

**TABLE 3:** Organic acid content in the cheeses (g/kg).

**Organic acids**	**LrC**	**StC**	**WcC**	**LhC**
Tartaric acid	65.630 ± 2.350^b^	93.200 ± 2.520^a^	63.800 ± 1.750^b^	67.560 ± 1.960^b^
Malic acid	10.530 ± 0.560^a^	10.980 ± 0.470^b^	6.780 ± 0.370^c^	15.950 ± 0.570^a^
Citric acid	0.443 ± 0.032^a^	0.393 ± 0.020^ab^	0.368 ± 0.024^b^	0.352 ± 0.036^b^
Lactic acid	19.209 ± 0.036^a^	9.144 ± 0.026^c^	14.910 ± 0.042^b^	2.906 ± 0.054^d^
Succinic acid	4.530 ± 0.019^a^	2.070 ± 0.024^b^	3.240 ± 0.021^ab^	3.540 ± 0.013^ab^
Pyruvic acid	0.009 ± 0.002^a^	0.005 ± 0.001^b^	0.003 ± 0.000^b^	0.003 ± 0.001^b^
Propionic acid	0.048 ± 0.001^a^	0.021 ± 0.002^b^	0.004 ± 0.001^c^	0.002 ± 0.001^c^
Total	100.399 ± 3.253^b^	115.813 ± 2.862^a^	89.105 ± 1.575^c^	90.313 ± 2.292^c^

*Data are expressed as the mean ± standard deviation from three replicate analyses (*n* = 3) of three replicate samples. The different lowercase letters in each row indicate a significant difference between the samples (*P* < 0.05).*

In all of the evaluated cheeses, the levels of tartaric acid, malic acid, lactic acid, and succinic acid were relatively high, whereas those of citric acid, pyruvic acid, and propionic acid were low, especially propionic acid and pyruvic acid. Among them, tartaric acid was the most abundant organic acid in all cheeses, especially in StC (93.20 g/kg). In addition to tartaric acid, high levels of lactic acid were also detected in LrC (19.21 g/kg) and in WcC (14.91 g/kg). The content of malic acid (15.95 g/kg) in LhC was higher than those in other cheeses. Among the four types of Kazak cheese assayed in this study, the total organic acid content in StC reached up to 115.81 g/kg, whereas only 89.11 g/kg was detected in WcC. The composition and relative content of organic acids in cheese obviously varied with the strains used to ferment the cheese (Ong and Shah, 2009; Güler, 2014).

### Texture of the Cheese

Texture is an important quality of cheese because different textures of cheese give people different taste perceptions. Cheeses can be divided into hard, semihard, and soft cheeses depending on the texture of the cheese (Rehman et al., 2018). To evaluate the effect of the strains on the texture of the cheeses, textural attributes of hardness, springiness, cohesiveness, chewiness, gumminess, and chewiness resilience of the Kazak cheese were detected.

**FIGURE 1:**
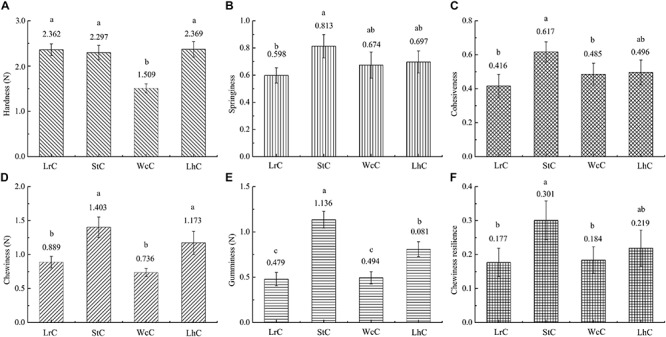
Comparative analysis of the textures of the cheeses. LrC, StC, WcC, and LhC represent cheeses fermented by *Lactobacillus rhamnosus* B10, *Streptococcus thermophilus* B8, *Weissella confusa* B14, and *Lactobacillus helveticus* B6, respectively. **(A–F)** Represent hardness, springiness, cohesiveness, chewiness, gumminess, and chewing resilience, respectively. The different lowercase letters in each figure represent significant differences between samples (*P* < 0.05).

The textures of WcC, LhC, StC, and LrC displayed significant differences (Figure 1). There was almost no significant difference (*P* < 0.05) in the hardness of the LrC, StC, and LhC cheeses, which varied inversely with the moisture percentage. Thus, the hardness of WcC agreed with the moisture percentage result previously described, and its high moisture made WcC relatively soft. The springiness, cohesiveness, gumminess, and chewiness resilience levels in WcC, LhC, and StC were higher than those in LrC. Interestingly, almost all of the textural attributes of StC were high, whereas those of WcC were low. It has been reported that changes in the textural properties of cheeses are affected by the acidification rate of the cheese during fermentation (Heller et al., 2003). An increased acidification rate is a prerequisite for the combination of casein and calcium, which makes cheese crisp. This result indicated that *S. thermophilus* B8 was appropriate for fermenting hard cheese because of its ability to produce a large amount of organic acids in a short time.

### Volatile Compounds in the Cheeses

Similar to other fermented foods, volatile compounds make cheese attractive. Alcohols, aldehydes, ketones, acids, and esters are the main volatile compounds in cheese, but the composition and relative content of volatile compounds clearly vary in cheese varieties (Güler, 2014; Cuffia et al., 2017). The volatile compounds in WcC, LhC, StC, and LrC were detected by the HS-SPME-GC-MS method. Twelve acids, 14 alcohols, 8 ketones, 16 esters, and 7 aldehydes were identified in the cheeses (Table 4), but the relative content of volatile compounds varied by strain. All of the cheeses, WcC, LhC, StC, and LrC, contained a high concentration of acetic acid and isoamyl acetate. In addition, the contents of 3-methylbutanoic acid, 2-methylpentanoic acid, and hexanoic acid in LrC; ethanol, 3-methylbutanoic acid, hexanoic acid, and ethyl caprylate in StC; and isoamylol, 3-methylbutanoic acid and ethyl acetate in WcC all reached up to 5%. With the exception of acetic acid and isoamyl acetate, other volatile compounds in LhC remained at a low level. Furthermore, all cheese samples contained a relatively high concentration of acetic acid, isobutyric acid, butanoic acid, 3-methylbutanoic acid, hexanoic acid, and isoamyl acetate, which indicated that volatile organic acids, including acetic acid, isobutyric acid, butanoic acid, 3-methylbutanoic acid, and hexanoic acid, significantly contributed to the sour taste in the cheeses. The acetic acid content in LhC was relatively high, which revealed that *L. helveticus* B6 could produce a high concentration of acetic acid (33.44%). In WcC, isoamyl acetate was the highest compound (23.98%), and acetic acid was rich both in LrC and StC.

**TABLE 4:** Relative concentration of the aroma components in the cheeses (%).

**Compound**	**Calculated RI**	**LrC**	**StC**	**WcC**	**LhC**
**Alcohols**					
Ethanol	1,482	2.117 ± 0.005^c^	7.004 ± 0.011^a^	3.253 ± 0.005^b^	0.103 ± 0.004^d^
Isobutanol	1,526	0.184 ± 0.004^c^	0.384 ± 0.005^b^	0.583 ± 0.005^a^	0.029 ± 0.007^d^
Isoamylol	1,142	–	4.253 ± 0.012^b^	8.758 ± 0.037^a^	1.615 ± 0.011^c^
Pentanol	1,265	3.942 ± 0.005^a^	0.433 ± 0.004^b^	0.387 ± 0.007^c^	0.093 ± 0.001^d^
Prenol	1,037	0.861 ± 0.001	–	0.207 ± 0.005	–
Hexanol	1,374	0.065 ± 0.005^d^	1.073 ± 0.002^a^	0.806 ± 0.004^c^	0.884 ± 0.011^b^
2-Nonen-1-ol	1,026	1.061 ± 0.003^a^	0.141 ± 0.009^d^	0.191 ± 0.008^b^	0.161 ± 0.007^c^
2-Ethylhexanol	974	0.279 ± 0.005^a^	0.084 ± 0.003^b^	0.037 ± 0.005^c^	0.037 ± 0.003^c^
1-Octanol	2,157	0.111 ± 0.002^d^	1.527 ± 0.004^a^	0.273 ± 0.002^b^	0.185 ± 0.006^c^
2,3-Butanediol	1,237	0.230 ± 0.002^d^	3.708 ± 0.011^a^	1.331 ± 0.005^c^	3.007 ± 0.013^b^
1-Nonanol	1,743	0.784 ± 0.004^a^	0.068 ± 0.003^d^	0.088 ± 0.006^c^	0.135 ± 0.004^b^
α-Cumyl alcohol	1,174	0.085 ± 0.003^a^	0.052 ± 0.005^b^	0.032 ± 0.003^c^	–
Phenylethyl alcohol	1,837	0.014 ± 0.003^a^	0.678 ± 0.007^b^	1.643 ± 0.011^a^	0.502 ± 0.004^c^
2-Methyloctan-3-ol	1,275	0.083 ± 0.002	0.040 ± 0.001	–	–
**Aldehydes**					
Hexanal	1,368	2.188 ± 0.005^b^	2.298 ± 0.005^a^	0.578 ± 0.004^d^	0.701 ± 0.008^c^
2-Heptenal	1,623	0.151 ± 0.003^c^	0.289 ± 0.002^b^	–	0.350 ± 0.006^a^
Nonanal	1,346	0.711 ± 0.002^b^	0.316 ± 0.003^d^	0.624 ± 0.004^c^	0.772 ± 0.008^a^
Decanal	1,573	–	0.295 ± 0.002^a^	0.084 ± 0.006^c^	0.117 ± 0.002^b^
Benzaldehyde	1,355	0.088 ± 0.001^b^	–	0.386 ± 0.007^a^	0.030 ± 0.001^c^
2-Nonenal	1,257	0.152 ± 0.002	–	–	0.421 ± 0.005
3-Butanolal	1,085	0.039 ± 0.001^a^	0.011 ± 0.004^c^	0.022 ± 0.002^b^	0.014 ± 0.003^c^
Octanal	1,548	–	0.052 ± 0.003^b^	0.036 ± 0.002^c^	0.208 ± 0.002^a^
**Acids**					
Acetic acid	1,758	29.364 ± 0.010^c^	31.262 ± 0.022^b^	22.844 ± 0.018^d^	33.436 ± 0.025^a^
Propanoic acid	1,029	0.068 ± 0.006^d^	1.093 ± 0.001^a^	0.303 ± 0.004^c^	0.341 ± 0.005^b^
Isobutyric acid	1,437	1.695 ± 0.002^d^	3.238 ± 0.007^b^	3.331 ± 0.009^a^	1.837 ± 0.007^c^
Butanoic acid	1,192	3.341 ± 0.006^c^	4.478 ± 0.015^b^	3.331 ± 0.006^c^	4.782 ± 0.009^a^
3-Methylbutanoic acid	1,090	7.047 ± 0.009^a^	5.765 ± 0.011^b^	5.653 ± 0.009^c^	2.765 ± 0.011^d^
2-Methylpentanoic acid	1,039	6.804 ± 0.006^a^	0.480 ± 0.008^c^	0.131 ± 0.006^d^	4.682 ± 0.011^b^
Hexanoic acid	964	5.174 ± 0.009^b^	6.333 ± 0.007^a^	4.277 ± 0.014^d^	4.651 ± 0.013^c^
Heptanoic acid	1,354	0.062 ± 0.000^b^	0.126 ± 0.004^a^	0.042 ± 0.002^d^	0.048 ± 0.001^c^
Octanoic acid	1,503	1.927 ± 0.006^a^	–	1.594 ± 0.009^c^	1.648 ± 0.004^b^
Nonanoic acid	1,374	0.062 ± 0.001^b^	0.089 ± 0.005^a^	0.057 ± 0.002^b^	0.027 ± 0.001^c^
Decanoic acid	1,097	0.375 ± 0.002^a^	0.332 ± 0.005^b^	0.278 ± 0.005^d^	0.325 ± 0.004^c^
Benzoic acid	1,275	0.692 ± 0.007^a^	0.447 ± 0.003^b^	0.408 ± 0.002^c^	0.308 ± 0.005^d^
**Esters**					
Ethyl acetate	1,379	1.734 ± 0.003^c^	2.691 ± 0.013^b^	5.761 ± 0.013^a^	0.881 ± 0.006^d^
Isobutyl acetate	1,027	–	0.183 ± 0.003	0.084 ± 0.002	–
Ethyl butanoate	1,768	0.602 ± 0.004	–	–	0.179 ± 0.002
Isoamyl acetate	1,942	12.424 ± 0.005^c^	8.643 ± 0.009^d^	23.981 ± 0.013^a^	18.575 ± 0.01^b^
Pentyl acetate	1,274	1.561 ± 0.001^b^	–	0.755 ± 0.003^c^	3.219 ± 0.013^a^
Ethyl hexanoate	1,354	0.420 ± 0.006^c^	0.580 ± 0.004^b^	0.494 ± 0.005^d^	2.091 ± 0.008^a^
Hexyl acetate	1,736	0.306 ± 0.002^b^	0.040 ± 0.001^d^	0.172 ± 0.002^c^	1.199 ± 0.009^a^
Ethyl L-lactate	1,932	0.397 ± 0.001^d^	0.959 ± 0.007^b^	1.493 ± 0.008^a^	0.758 ± 0.006^c^
Heptyl acetate	1,093	0.130 ± 0.001^b^	0.147 ± 0.006^a^	0.046 ± 0.002^c^	0.152 ± 0.001^a^
Ethyl caprylate	1,127	5.428 ± 0.003^a^	0.433 ± 0.005^c^	0.422 ± 0.003^d^	3.354 ± 0.001^b^
Octyl acetate	1,425	0.069 ± 0.002	–	–	0.025 ± 0.003
Butyrolactone	1,237	–	0.052 ± 0.000	–	0.050 ± 0.003
Ethyl caprate	1,358	–	–	–	0.385 ± 0.005
Phenethyl acetate	1,642	1.369 ± 0.003^c^	0.334 ± 0.002^d^	2.301 ± 0.010^b^	3.820 ± 0.007^a^
5-Decanolide	1,138	0.060 ± 0.001^b^	0.332 ± 0.005^a^	0.027 ± 0.002^d^	0.042 ± 0.005^c^
1,3-Diacetoxypropane	1,029	–	0.032 ± 0.002	–	0.024 ± 0.003
**Ketones**					
Acetol	1,528	0.013 ± 0.000^c^	0.072 ± 0.003^b^	1.511 ± 0.011^a^	–
5-Methyl-2-hexanone	1,069	1.979 ± 0.002^b^	4.450 ± 0.011^a^	0.648 ± 0.003^c^	0.102 ± 0.002^d^
2-Heptanone	1,463	0.675 ± 0.002^a^	0.674 ± 0.006^a^	0.021 ± 0.001^c^	0.364 ± 0.004^b^
Acetoin	1,137	–	2.488 ± 0.003^a^	0.136 ± 0.002^c^	0.318 ± 0.001^b^
6-Methylhept-5-en-2-one	1,265	–	0.278 ± 0.001^b^	0.135 ± 0.001^c^	0.159 ± 0.002^a^
2-Nonanone	1,386	1.213 ± 0.003^a^	1.127 ± 0.006^b^	0.391 ± 0.001^c^	0.090 ± 0.003^d^
2-Undecanone	963	0.085 ± 0.000^b^	0.140 ± 0.004^a^	0.055 ± 0.003^c^	–
3-methyl-2-hexanone	976	–	–	0.004 ± 0.001	–

*Data are expressed as the mean ± standard deviation from three replicate analyses (*n* = 3) of three replicate samples. The different lowercase letters in each row indicate a significant difference between the samples (*P* < 0.05). LrC, StC, WcC, and LhC represent samples collected at the fifth stage. The symbol “–” indicates that the compound was not detected.*

**FIGURE 2:**
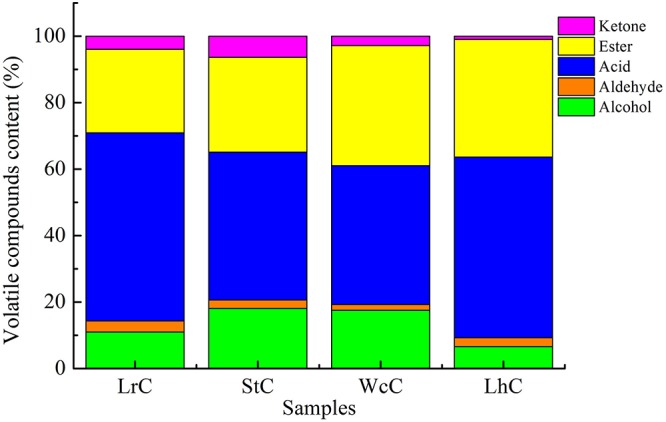
Volatile compounds in the cheeses.

Acids and esters were the main volatile compounds after the fermentation of WcC, LhC, LrC, and StC (Figure 2). Under the effects of proteases, lipases, and lactases, cheese could accumulate a high concentration of amino acids, fatty acids, and organic acids, which participated in various reactions to generate a unique taste and aroma in the cheese. We found that this may be due to FAA catabolism and lactose and citric acid metabolism, which can promote the synthesis of acidic compounds, such as acetic acid. Esters can be synthesized in two ways: the esterification reaction between alcohol and carboxylic acid and the alcoholysis reaction between alcohol and acyl glycerol (Cuffia et al., 2017). The formation of aldehydes, such as benzaldehyde, begins with the conversion of Phe by aminotransferases to phenylpyruvate, which is then converted to benzaldehyde by chemical oxidation (Bian et al., 2016). The formation of ketones is related to the activity of lipases on acyl lipids, which releases fatty acids, and the catabolism of flavor substances through the β-oxidation pathway (Collins et al., 2003).

### Dynamic Changes in the Volatile Compounds in the Cheeses

**FIGURE 3:**
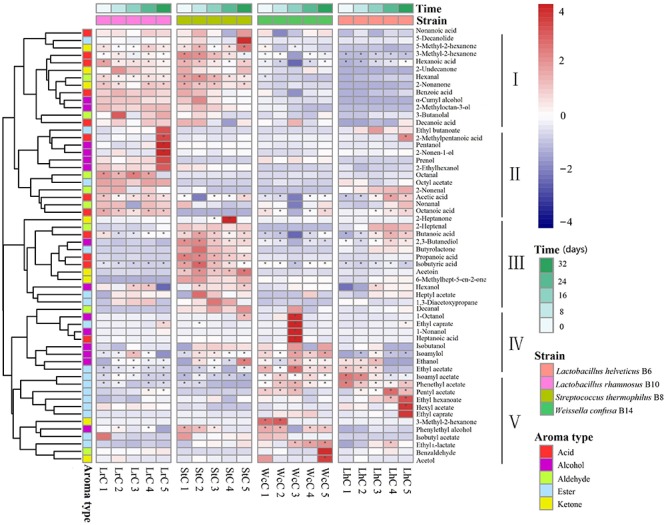
Heatmap analysis of five fermentation periods of the cheeses. LrC, StC, WcC, and LhC represent cheeses fermented by *Lactobacillus rhamnosus* B10, *Streptococcus thermophilus* B8, *Weissella confusa* B14, and *Lactobacillus helveticus* B6, respectively. The five fermentation times were 0, 8, 16, 24, and 32 days, respectively. The asterisk indicates the substance with content greater than 1 in the figure.

To better understand the role of the enzyme-producing LABs in Kazak cheese in the post-fermentation ripening process, we compared the composition and changes in the flavor components during the maturing process of Kazak cheese fermented by different strains (Figure 3).

According to the flavor clustering results, the volatile compounds were divided into five categories based on trends observed throughout the ripening process: and contained 13, 12, 12, 9, and 12 aromatic compounds, respectively. In LrC, the flavor component between categories I and II was the largest of the five categories, and the content increased gradually. Categories I, III, and IV in StC were the largest groups, whereas categories IV and V changed significantly in WcC. However, few differences in the contents of flavor substances were observed in LhC. Volatile compounds with content greater than 1% were marked with an asterisk in Figure 3 and constantly changed during the fermentation process. The main compounds in LrC were 2-methylpentanoic acid, pentanol, and 2-nonen-1-ol, whereas 2-heptanone was most abundant in StC during fermentation. The main compounds in WcC were 1-octanol, ethyl caprylate, 1-nonanol, and heptanoic acid, whereas the crucial flavor compounds in LhC were ethyl hexanoate and hexyl acetate. This indicates that the metabolic changes in the volatile compounds generated during fermentation were closely associated with the added LAB starter.

**FIGURE 4:**
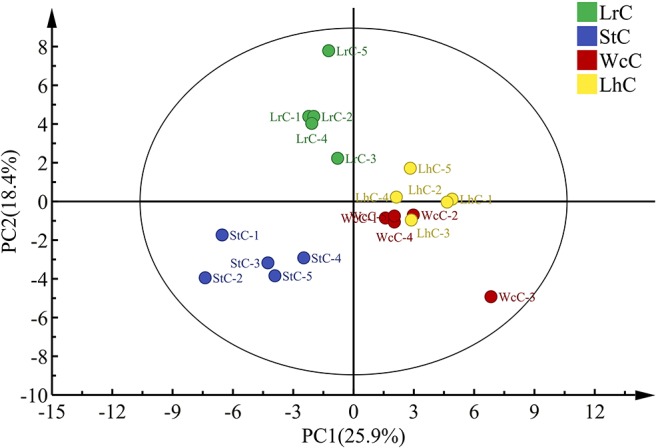
Principal component analysis of the volatile compounds in the cheeses. LrC, StC, WcC, and LhC represent cheeses fermented by *Lactobacillus rhamnosus* B10, *Streptococcus thermophilus* B8, *Weissella confusa* B14, and *Lactobacillus helveticus* B6, respectively. The five fermentation times were 0, 8, 16, 24, and 32 days, respectively.

Principal component analysis is the most widely used multivariate statistical tool in data analysis. The interpretation rates of PC1 and PC2 were 25.9 and 18.4%, respectively, and the sum of PC1 and PC2 was 44.3%. Based on the PCA plot, the four cheese samples fermented by different strains were distinguished from each other (Figure 4). In addition to the third fermentation period of LhC, both WcC and LhC, fermented by *W. confusa* B14 and *L. helveticus* B6, respectively, were highly similar, which may be related to the production of proteases and lipases by the two strains.

**FIGURE 5:**
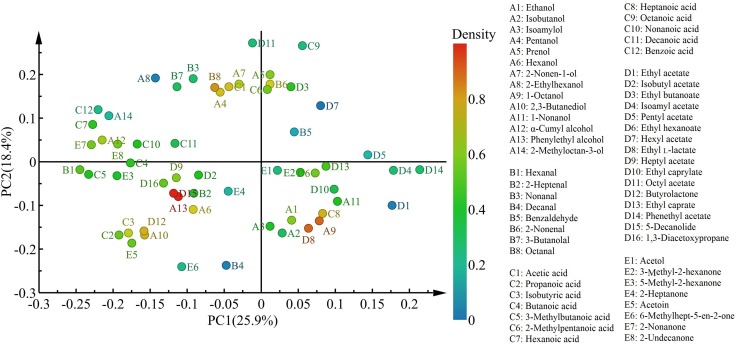
Characteristic flavors in the four cheeses after fermentation. LrC, StC, WcC, and LhC represent cheeses fermented by *Lactobacillus rhamnosus* B10, *Streptococcus thermophilus* B8, *Weissella confusa* B14, and *Lactobacillus helveticus* B6, respectively.

With respect to the distribution of flavor compounds in the four cheese samples (Figure 5), in the left quadrant, LrC varied positively with pentanol, 2-nonen-1-ol, octanal, and acetic acid, while StC was highly interrelated with hexanol, 2,3-butanediol, phenylethyl alcohol, isobutyric acid, butyrolactone, and 5-decanolide. In the right quadrant, 1-octanol, heptanoic acid, ethyl L-lactate, and 2-nonenal had a significant effect on the flavor of WcC and LhC. Among them, phenylethyl alcohol and 5-decanolide were the most abundant compounds in StC with a high correlation coefficient (mainly up to 0.80).

### Construction of Volatile Compound Fingerprints for the LABs

**FIGURE 6:**
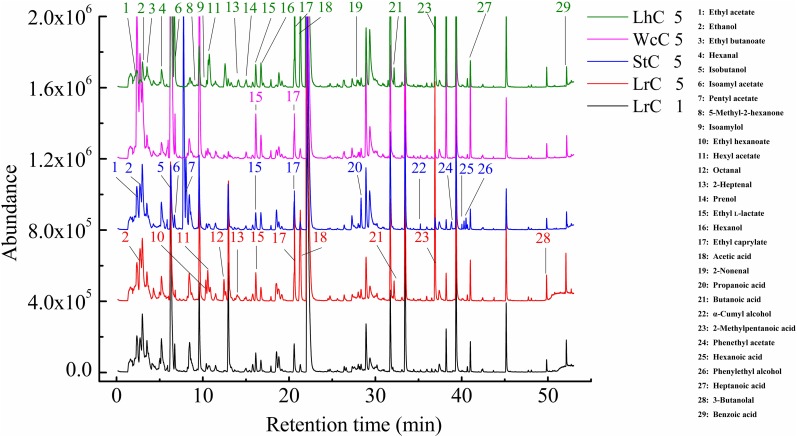
Construction of volatile compound fingerprints for the lactic acid bacteria.

The differences among the types of flavors could be attributed to the variations in the LABs used to make cheese (Supplementary Figure S3). The contents of ethanol, ethyl hexanoate, hexyl acetate, ethyl L-lactate, ethyl caprylate, acetic acid, 2-methylpentanoic acid, and 3-butanolal increased at the end of fermentation process compared to those observed during early stage of fermentation in LrC, whereas octanal, 2-heptenal, and butanoic acid gradually formed in the late stage of fermentation and were not detected during the early stage of fermentation process. *L. rhamnosus* B10 particularly contributed to the formation of ethanol, hexyl acetate, ethyl caprylate, acetic acid, and 2-methylpentanoic acid (Figure 6). In StC, the levels of ethyl acetate, ethanol, ethyl L-lactate, ethyl caprylate, propanoic acid, and α-cumyl alcohol increased, whereas those of isobutanol and isoamyl acetate decreased. In addition, pentyl acetate, phenethyl acetate, hexanoic acid, and phenylethyl alcohol were not detected in the early stage of fermentation. It could be concluded that *S. thermophilus* B8 was mostly beneficial to the formation of ethyl acetate, ethanol, and ethyl L-lactate. In WcC, which was fermented by *W. confusa* B14, the variation in the levels of flavor compounds was low, and only the contents of ethyl butanoate, ethyl L-lactate, and ethyl caprylate increased. In LrC, hexanal, isoamyl acetate, hexyl acetate, prenol, ethyl L-lactate, hexanol, ethyl caprylate, acetic acid, 2-methylpentanoic acid, heptanoic acid, and benzoic acid were enhanced by *L. helveticus* B6, whereas 2-heptenal and butanoic acid gradually formed at the end of fermentation. However, ethyl acetate, ethanol, ethyl butanoate, and 2-nonenal decreased during fermentation, whereas 5-methyl-2-hexanone and isoamylol were consumed (Supplementary Table S3). These results suggested that the compounds and contents during metabolism were different and closely related to the types and activities of enzymes present.

## Discussion

Proteolysis, lipolysis, and lactose decomposition were key factors in the formation of flavor precursor compounds and texture improvements in cheese (Ozcan and Kurdal, 2012; Mörschbächer et al., 2016; Ozturkoglu-Budak et al., 2016). Among the four types of cheeses, the contents of TFAA and Glu were the highest in WcC, which are extremely related to the high protease activity of *W. confusa* B14. Besides, all cheeses had a high level of Glu, which is consistent with the results of previous studies (Hou et al., 2014). The four types of cheese were all rich in tartaric acid, lactic acid, and malic acid, which could be used as preservatives in cheese fermentation. Because organic acids diffused in bacterial cell membranes, dissociated in the cytoplasm, reduced intracellular pH, and led to cessation of growth or cell death, the synergies of organic acids could produce antibacterial properties to inhibit the contamination by molds and yeasts (Kisadere et al., 2018). Furthermore, the texture of StC fermented by *S. thermophilus* B8 was more consistent with the characteristics of Kazak cheese than the cheese fermented by other LABs.

Among the four types of cheese, acids and esters account for a large proportion, which could be produced by AA catabolism, lactose metabolism, citrate metabolism, esterification reaction, and alcoholysis reaction (Liu et al., 2004; Cuffia et al., 2017). The concentrations of volatile compounds are shown in Supplementary Table S4. Aroma analysis showed that the banana aroma resulted by isoamyl acetate was highest in all cheese samples (Table 5). Except for isoamyl acetate, high OAVs^2^ were also calculated for ethyl acetate (107.79) that were orange smelling and nonanal (53.07) with fatty, herbaceous, green, and sweet note quality in WcC (Supplementary Figure S4). Furthermore, ethyl acetate with orange odor, hexanal with green odor, and ethyl hexanoate with brandy, orange, and sour odor notes in StC had OAVs of 22.30, 19.04, and 10.92, respectively. OAVs higher than 1 was also determined for hexanol, 2,3-butanediol, octanal, ethyl butanoate, 3-methylbutanoic acid, and 2-undecanone in different cheeses. As the lipases of LABs were released, the fatty acids formed by lipolysis were first oxidized to α-ketoacids via the β-oxidation pathway and then were decarboxylated by carboxyl groups to their corresponding methyl ketones, which is how 2-undecanone, 2-heptanone, and 2-nonanone formed (Van Mastrigt et al., 2018). In total, volatile compounds with aroma smell could be divided into eight types. Among which fruit flavor was the strongest aroma in the four cheeses. In addition, odors with herbaceous, fatty, brandy, pungent, and sweet flavors also constituted the primary aroma types of Kazak cheese, with the aroma of WcC being the most prominent.

**TABLE 5:** Concentrations, odor descriptions, and threshold values of volatiles detected in different cheeses (μg/kg of sample).

**Compound**	**Odor description**	**Threshold in water (μg/L)**	**OAV in LrC**	**OAV in StC**	**OAV in WcC**	**OAV in LhC**
Hexanol	Herbaceous, green	5.6	–	7.94	13.46	3.56
2,3-Butanediol	Fruity, onion	95.1	–	1.62	1.31	0.71
Hexanal	Green	5	3.67	19.04	10.81	3.16
Nonanal	Herbaceous, green, fatty, sweet	1.1	5.42	11.90	53.07	15.82
Octanal	Sweet, citrus, fatty, pungent, green	0.587	–	3.67	5.74	7.99
3-Methylbutanoic acid	Cheesy, pungent, sweet	500	–	–	1.06	–
Ethyl acetate	Fruity, orange	5	2.91	22.30	107.79	3.97
Ethyl butanoate	Fruity, apple	0.9	5.61	–	–	4.48
Isoamyl acetate	Fruity, banana	0.15	694.08	2,387.20	14,956.15	2,791.21
Ethyl hexanoate	Brandy, orange, sour	2.2	1.60	10.92	21.00	21.42
2-Undecanone	Floral, tallow	5.5	–	1.05	–	–

*The symbol “–” indicates that the OAV of compound was less than one. The threshold values in water were according to Gemert (2011).*

Considering the differences in the composition and content of the flavor compounds in the cheese fermented by a single strain of bacteria, whether the cofermentation of the strains will also promote the formation of the characteristic flavors of the cheese is worth exploring. All LABs used to ferment Kazak cheese require salt-tolerant properties to play a role in cheese ripening. This is related to the presence of a glycine-betaine transporter in the genomes of the strains (Kazou et al., 2018). Therefore, special gene fragments present in the LABs associated with cheese flavor can be further analyzed in subsequent experiments.

## Conclusion

The results of this study showed the contributions of selected LABs from Kazak cheese with protease, lipase, or β-galactosidase activities on cheese quality and flavor. The results revealed that all the assayed cheeses had a high level of Glu and tartaric acid. *S. thermophilus* B8 contributed to the formation of organic acids, whereas *W. confusa* B14 promoted the production of amino acids in WcC. Cheese made with *S. thermophilus* B8 had ideal texture properties, including high springiness, cohesiveness, chewiness, gumminess, and chewiness resilience. The differences between the volatile compounds in cheese suggested that the LABs used in cheese fermentation greatly affected the flavor of cheese. Odor analysis showed that fruit flavor was the strongest aroma in the four cheeses. This is because the four LABs produced a high concentration of isoamyl acetate. In addition, WcC was accompanied by strong herbaceous, sweet, and fatty aromas.

## Data Availability Statement

The sequencing data can be found in NCBI under the following accession numbers: MN966848, MN966849, MN966850, and MN966851.

## Author Contributions

JL performed the experiment, collected the test data, and drafted the manuscript. QH processed the data, XZ provided some data for the study. ZG and YC made the graphs and tables. XS and BW designed the study and revised the manuscript. KL and DZ submitted amendments to the manuscript.

## Conflict of Interest

The authors declare that the research was conducted in the absence of any commercial or financial relationships that could be construed as a potential conflict of interest.

## Footnotes

1 https://blast.ncbi.nlm.nih.gov/Blast.cgi

2 Definition of OAVs: divide the concentration of each compound by the odor threshold in water as reported in the literature.
