# Supplementary material for: Investigation of the Lactic Acid Bacteria in Kazak Cheese and Their Contributions to Cheese Fermentation
Source: Front Microbiol. 2020 Mar 12;11:228. doi: 10.3389/fmicb.2020.00228 (PMC7080652; doi:10.3389/fmicb.2020.00228)
Supplement: Supplementary file 1 [file Data_Sheet_1.docx]

Supplementary materials for:

**Investigation of The Lactic Acid Bacteria in Kazak Cheese and Their Contributions to Cheese Fermentation**

**Jie Li^┼^, Qian Huang^┼^, Xiaochun Zheng****, Zhengkai Ge, Ke Lin, Dandan Zhang, Yu Chen,** **Bin Wang****^*^, Xuewei Shi^*^**

Food College, Shihezi University, Shihezi 832000, Xinjiang Uygur Autonomous Region, P. R. China.

* Corresponding authors

E-mail addresses: B. W.: [binwang0228@shzu.edu.cn](mailto:binwang0228@shzu.edu.cn); X. S.: [shixuewei@shzu.edu.cn](mailto:shixuewei@shzu.edu.cn)

Tel.: 86-0993-2058093

^┼^ These authors contributed equally to this work.

Catalog

[Supplementary tables 1](#_Toc10294)

[Table S1. Similarity alignment of gene sequence 1](#_Toc14106)

[Table S2. Identification and enzyme activity of LAB (U/mL) 2](#_Toc9057)

[Table S3. Construction of fingerprints of volatile compounds in cheeses 3](#_Toc6760)

[Table S4. Concentrations of volatile compounds in cheeses (µg/kg) 4](#_Toc11699)

[Supplementary figures 6](#_Toc18140)

[Figure S1. Hydrolysis halo of selected strains 6](#_Toc3501)

[Figure S2. Numbers of viable bacteria in cheeses at different fermentation periods (cfu/kg) 7](#_Toc8199)

[Figure S3. Construction of volatile compound fingerprints for the LABs 8](#_Toc7042)

[Figure S4. Radar analysis of flavor compounds 9](#_Toc7335)

# Supplementary tables

## **Table S1.** Similarity alignment of gene sequence

| Strain | Size (bp) | Identification | Similarity (%) | Accession number |
| --- | --- | --- | --- | --- |
| B1 | 1,059 | *Lactobacillus casei* strain NWAFU1544 | 97.68 | MG551218.1 |
| B2 | 1,151 | *Lactobacillus casei* strain NWAFU1544 | 98.68 | MG551218.1 |
| B3 | 1,252 | *Lactobacillus casei* strain NWAFU1575 | 96.88 | MG551255.1 |
| B4 | 1,243 | *Lactobacillus casei* strain NWAFU1544 | 96.76 | MG551249.1 |
| B5 | 1,233 | *Lactobacillus paracasei* strain 05 | 97.03 | JN560834.1 |
| B6 | 1,237 | *Lactobacillus helveticus* strain hstb-6 | 97.83 | KX822707.1 |
| B7 | 1,153 | *Lactobacillus helveticus* strain LH5 | 97.28 | CP019581.1 |
| B8 | 1,102 | *Streptococcus thermophilus* strain N4L | 99.73 | LS974444.1 |
| B9 | 1,080 | *Streptococcus thermophilus* strain ST3 | 99.26 | MG815652.1 |
| B10 | 1,019 | *Lactobacillus rhamnosus* strain A5 | 98.41 | MK329243.1 |
| B11 | 1,250 | *Lactococcus lactis* strain Sourdough M5 | 97.39 | MG754635.1 |
| B12 | 1,222 | *Lactobacillus rhamnosus* strain 8 | 97.7 | MN030350.1 |
| B13 | 1,226 | *Streptococcus thermophilus* strain N4L | 97.32 | LS974444.1 |
| B14 | 1,226 | *Weissella confusa* strain RCB331 | 97.19 | KT260543.1 |
| B15 | 1,219 | *Streptococcus thermophilus* strain KLDS 3.0606 | 98.84 | EU419603.1 |
| B16 | 1,051 | *Weissella confusa* strain FB054 | 96.51 | MF945623.1 |
| B17 | 1,178 | *Lactococcus lactis* strain Sourdough M8 | 98.16 | MG754628.1 |
| B18 | 1,160 | *Lactococcus gravieae* strain def2 | 99.10 | MH198321.1 |
| B19 | 1,169 | *Leuconostoc lactis* strain A4584 | 98.80 | MN441494.1 |
| B20 | 1,211 | *Leuconostoc lactis* strain KLDS 5.0604 | 98.75 | EU419606.1 |
| B21 | 1,217 | *Leuconostoc lactis* strain A4584 | 97.81 | MN441494.1 |
| B22 | 1,050 | *Lactobacillus plantarum* strain CP2 | 99.52 | MN244500.1 |

## **Table S2.** Identification and enzyme activity of LAB (U/mL)

| Identification | Number | Protease activity | Lipase activity | β-galactosidase activity |
| --- | --- | --- | --- | --- |
| *Streptococcus thermophilus* | B8 | 124 ± 1.32 | 87 ± 1.13 | 162 ± 1.21 |
| *Weissella confusa* | B14 | 139 ± 1.03 | 96 ± 1.06 | — |
| *Lactobacillus helveticus* | B6 | 103 ± 0.94 | 115 ± 1.65 | — |
| *Lactococcus rhamnosus* | B10 | 120 ± 1.25 | — | 186 ± 1.17 |
| *Leuconostoc lactis* | B19 | 87 ± 0.87 | — | 157 ± 1.35 |
| *Lactobacillus paracasei* | B5 | 113 ± 1.06 | — | — |
| *Leuconostoc lactis* | B20 | 105 ± 0.95 | — | — |
| *Lactobacillus casei* | B4 | 96 ± 0.78 | — | — |
| *Lactobacillus helveticus* | B7 | — | 83 ± 0.88 | 106 ± 1.18 |
| *Lactobacillus casei* | B1 | 86 ± 0.88 | 94 ± 0.82 | 138 ± 1.33 |
| *Leuconostoc lactis* | B21 | 102 ± 1.14 | — | — |
| *Weissella confusa* | B16 | 98 ± 0.92 | 87 ± 0.91 | — |
| *Streptococcus thermophilus* | B9 | 118 ± 2.03 | 68 ± 0.76 | — |
| *Streptococcus thermophilus* | B13 | 102 ± 1.06 | 73 ± 0.95 | — |
| *Streptococcus thermophilus* | B15 | 116 ± 1.31 | — | 140 ± 1.67 |
| *Lactococcus lactis* | B11 | 113 ± 1.48 | 77 ± 0.85 | — |
| *Lactobacillus casei* | B3 | — | — | 158 ± 1.42 |
| *Lactobacillus casei* | B2 | 96 ± 0.92 | 87 ± 0.97 | 155 ± 1.75 |

^1^ Symbol “—” stands for undetectable.

^2^ Data is expressed as the mean ± standard deviation and three replicate analyses (n = 3) of three replicate samples.

## **Table S3.** Construction of fingerprints of volatile compounds in cheeses

| Number | Flavor compounds | Time (min) | LrC-5 | StC | WcC | LhC |
| --- | --- | --- | --- | --- | --- | --- |
| 1 | Ethyl acetate | 2.36 |  | 10.028* |  | −0.909 |
| 2 | Ethanol | 2.73 | 8.140* | 38.015* |  | −0.981 |
| 3 | Ethyl butanoate | 4.25 |  |  | 1.278 | −0.743 |
| 4 | Hexanal | 5.20 |  |  |  | 1.424 |
| 5 | Isobutanol | 5.91 |  | −0770 |  |  |
| 6 | Isoamyl acetate | 6.25 |  | −0.474 |  | 6.954* |
| 7 | Pentyl acetate | 7.81 |  | +1 |  |  |
| 8 | 5-Methyl-2-hexanone | 8.24 |  |  |  | −1 |
| 9 | Isoamylol | 9.63 |  |  |  | −1 |
| 10 | Ethyl hexanoate | 10.56 | 1.912 |  |  |  |
| 11 | Hexyl acetate | 12.47 | 16.959* |  |  | 2.134 |
| 12 | Octanal | 13.156 | +1 |  |  |  |
| 13 | 2-Heptenal | 14.85 | +1 |  |  | +1 |
| 14 | Prenol | 15.00 |  |  |  | 1.492 |
| 15 | Ethyl l-lactate | 16.15 | 2.942 | 4.069* | 1.091 | 2.490 |
| 16 | Hexanol | 16.74 |  |  |  | 4.495* |
| 17 | Ethyl caprylate | 21.29 | 7.421* | 1.532 | 3.783 | 6.954* |
| 18 | Acetic acid | 22.06 | 16.137* |  |  | 8.725* |
| 19 | 2-Nonenal | 26.59 |  |  |  | −0.862 |
| 20 | Propanoic acid | 27.26 |  | 2.835 |  |  |
| 21 | Butanoic acid | 32.26 | +1 |  |  | +1 |
| 22 | α-Cumyl alcohol | 36.52 |  | 1.984 |  |  |
| 23 | 2-Methylpentanoic acid | 36.93 | 62.935* |  |  | 32.762* |
| 24 | Phenethyl acetate | 38.2 |  | +1 |  |  |
| 25 | Hexanoic acid | 40.66 |  | +1 |  |  |
| 26 | Phenylethyl alcohol | 41.00 |  | +1 |  |  |
| 27 | Heptanoic acid | 42.42 |  |  |  | 1.203 |
| 28 | 3-Butanolal | 50.80 | 1.445 |  |  |  |
| 29 | Benzoic acid | 52.14 |  |  |  | 1.871 |

^1^ Symbol "+1" and "−1" indicated compounds that are formed and disappeared in the later stages of fermentation.

^2^ The data was calculated as (late peak area − initial peak area) / initial peak area.

^3^ Symbol "*" indicated that the compound changes significantly.

## **Table S4.** Concentrations of volatile compounds in cheeses (µg/kg)

| Compounds | RI | LrC | StC | WcC | LhC |
| --- | --- | --- | --- | --- | --- |
| **Alcohols** |  |  |  |  |  |
| Ethanol | 1,482 | 17.740 ± 4.190^b^ | 290.176 ± 45.573^a^ | 304.318 ± 46.775^a^ | 2.322 ± 0.902^b^ |
| Isobutanol | 1,526 | 1.542 ± 0.335^c^ | 15.909 ± 2.072^b^ | 54.540 ± 4.678^a^ | 0.654 ± 0.078^c^ |
| Isoamylol | 1,142 | — | 176.202 ± 4.972^b^ | 819.311 ± 34.614^a^ | 36.402 ± 2.479^c^ |
| Pentanol | 1,265 | 33.034 ± 0.419^a^ | 17.939 ± 1.657^b^ | 36.204 ± 6.549^a^ | 2.096 ± 0.225^d^ |
| Prenol | 1,037 | 7.215 ± 0.647 | — | 19.365 ± 4.678 | — |
| Hexanol | 1,374 | 0.545 ± 0.042^d^ | 44.454 ± 0.829^b^ | 75.401 ± 3.742^a^ | 19.925 ± 2.479^c^ |
| 2-Nonen-1-ol | 1,026 | 8.891 ± 0.251^b^ | 5.842 ± 0.373^c^ | 17.868 ± 2.748^a^ | 3.629 ± 0.158^c^ |
| 2-Ethylhexanol | 974 | 2.338 ± 0.419^b^ | 3.480 ± 0.043^a^ | 3.461 ± 0.047^a^ | 0.834 ± 0.008^c^ |
| 1-Octanol | 2,157 | 0.930 ± 0.017^d^ | 63.264 ± 1.657^a^ | 25.539 ± 1.871^b^ | 4.170 ± 0.135^c^ |
| 2,3-Butanediol | 1,237 | 1.927 ± 0.168^d^ | 153.622 ± 7.557^a^ | 124.515 ± 6.874^b^ | 67.778 ± 2.930^c^ |
| 1-Nonanol | 1,743 | 6.570 ± 0.335^b^ | 2.817 ± 0.124^c^ | 8.232 ± 0.561^a^ | 3.043 ± 0.902^c^ |
| α-Cumyl alcohol | 1,174 | 0.712 ± 0.025^c^ | 2.154 ± 0.207^b^ | 2.994 ± 0.281^a^ | — |
| Phenylethyl alcohol | 1,837 | 0.117 ± 0.021^d^ | 28.090 ± 2.900^b^ | 153.703 ± 10.291^a^ | 11.315 ± 0.902^c^ |
| 2-Methyloctan-3-ol | 1,275 | 0.696 ± 0.168 | 1.657 ± 0.414 | — | — |
| **Aldehydes** |  |  |  |  |  |
| Hexanal | 1,368 | 18.335 ± 4.190^c^ | 95.206 ± 2.072^a^ | 54.072 ± 3.742^b^ | 15.801 ± 1.803^d^ |
| 2-Heptenal | 1,623 | 1.265 ± 0.251^c^ | 11.973 ± 0.829^a^ | — | 7.889 ± 1.352^b^ |
| Nonanal | 1,346 | 5.958 ± 0.168^d^ | 13.092 ± 1.243^c^ | 58.375 ± 3.742^a^ | 17.401 ± 1.803^b^ |
| Decanal | 1,573 | — | 12.222 ± 0.829^a^ | 7.858 ± 0.561^b^ | 2.637 ± 0.451^c^ |
| Benzaldehyde | 1,355 | 0.737 ± 0.084^b^ | — | 36.110 ± 1.054^a^ | 0.676 ± 0.025^b^ |
| 2-Nonenal | 1,257 | 1.274 ± 0.168 | — | — | 9.489 ± 1.127 |
| 3-Butanolal | 1,085 | 0.327 ± 0.024^b^ | 0.456 ± 0.066^b^ | 2.058 ± 0.187^a^ | 0.316 ± 0.007^b^ |
| Octanal | 1,548 | — | 2.154 ± 0.124^c^ | 3.368 ± 0.187^b^ | 4.688 ± 0.451^a^ |
| **Acids** |  |  |  |  |  |
| Acetic acid | 1,758 | 246.070 ± 8.38^d^ | 1295.185 ± 91.146^b^ | 2137.056 ± 168.390^a^ | 753.647 ± 56.350^c^ |
| Propanoic acid | 1,029 | 0.570 ± 0.050^d^ | 45.283 ± 5.414^a^ | 28.346 ± 3.742^b^ | 7.686 ± 1.127^c^ |
| Isobutyric acid | 1,437 | 14.204 ± 0.168^d^ | 134.150 ± 2.900^b^ | 311.615 ± 8.420^a^ | 41.406 ± 1.578^c^ |
| Butanoic acid | 1,192 | 27.998 ± 5.028^d^ | 185.524 ± 6.215^b^ | 311.615 ± 5.613^a^ | 107.786 ± 2.029^c^ |
| 3-Methylbutanoic acid | 1,090 | 59.054 ± 7.542^c^ | 238.844 ± 4.557^b^ | 528.838 ± 8.420^a^ | 62.323 ± 2.479^c^ |
| 2-Methylpentanoic acid | 1,039 | 57.018 ± 0.503^b^ | 19.886 ± 3.314^c^ | 12.255 ± 0.561^d^ | 105.532 ± 2.479^a^ |
| Hexanoic acid | 964 | 43.358 ± 0.754^d^ | 262.376 ± 2.900^b^ | 400.113 ± 13.097^a^ | 104.834 ± 2.930^c^ |
| Heptanoic acid | 1,354 | 0.520 ± 0.024^d^ | 5.320 ± 0.166^a^ | 3.929 ± 0.157^b^ | 1.082 ± 0.225^c^ |
| Octanoic acid | 1,503 | 16.148 ± 0.503^c^ | — | 149.119 ± 8.420^a^ | 37.146 ± 2.902^b^ |
| Nonanoic acid | 1,374 | 0.520 ± 0.084^b^ | 3.687 ± 0.207^a^ | 5.332 ± 1.871^a^ | 0.609 ± 0.023^b^ |
| Decanoic acid | 1,097 | 3.143 ± 0.154^d^ | 13.755 ± 2.041^b^ | 26.007 ± 1.678^a^ | 7.326 ± 1.356^c^ |
| Benzoic acid | 1,275 | 5.799 ± 0.587^c^ | 18.519 ± 1.249^b^ | 38.168 ± 1.878^a^ | 6.942 ± 1.127^c^ |
| **Esters** |  |  |  |  |  |
| Ethyl acetate | 1,379 | 14.531 ± 2.514^c^ | 111.488 ± 5.386^b^ | 538.942 ± 12.162^a^ | 19.858 ± 1.352^c^ |
| Isobutyl acetate | 1,027 | — | 7.582 ± 1.243 | 7.858 ± 1.871 | — |
| Ethyl butanoate | 1,768 | 5.045 ± 0.335 | — | — | 4.035 ± 0.451 |
| Isoamyl acetate | 1,942 | 104.113 ± 4.190^c^ | 358.079 ± 37.287^b^ | 2243.423 ± 121.615^a^ | 418.681 ± 24.743^b^ |
| Pentyl acetate | 1,274 | 13.081 ± 1.642^b^ | — | 70.630 ± 2.807^a^ | 72.556 ± 2.930^a^ |
| Ethyl hexanoate | 1,354 | 3.520 ± 0.503^c^ | 24.029 ± 1.657^b^ | 46.214 ± 4.678^a^ | 47.131 ± 1.803^a^ |
| Hexyl acetate | 1,736 | 2.564 ± 0.168^c^ | 1.657 ± 0.414^c^ | 16.091 ± 1.459^b^ | 27.025 ± 2.029^a^ |
| Ethyl l-lactate | 1,932 | 3.327 ± 0.526^d^ | 39.731 ± 2.9^b^ | 139.670 ± 7.484^a^ | 17.085 ± 1.352^c^ |
| Heptyl acetate | 1,093 | 1.089 ± 0.078^d^ | 6.090 ± 0.249^a^ | 4.303 ± 0.574^b^ | 3.426 ± 0.225^c^ |
| Ethyl caprylate | 1,127 | 45.487 ± 2.541^b^ | 17.939 ± 2.072^d^ | 39.478 ± 2.807^c^ | 75.599 ± 2.254^a^ |
| Octyl acetate | 1,425 | 0.578 ± 0.168 | — | — | 0.564 ± 0.068 |
| Butyrolactone | 1,237 | — | 2.154 ± 0.034 | — | 1.127 ± 0.676 |
| Ethyl caprate | 1,358 | — | — | — | 8.678 ± 1.127 |
| Phenethyl acetate | 1,642 | 11.472 ± 2.136^c^ | 13.838 ± 3.362^c^ | 215.259 ± 9.355^a^ | 86.103 ± 15.778^b^ |
| 5-Decanolide | 1,138 | 0.503 ± 0.059^c^ | 13.755 ± 1.015^a^ | 2.526 ± 0.146^b^ | 0.947 ± 0.113^c^ |
| 1,3-Diacetoxypropane | 1,029 | — | 1.326 ± 0.083 | — | 0.541 ± 0.088 |
| **Ketones** |  |  |  |  |  |
| Acetol | 1,528 | 0.109 ± 0.004^b^ | 2.983 ± 0.124^b^ | 141.354 ± 10.107^a^ | — |
| 3-Methyl-2-hexanone | 976 | — | — | 0.164 ± 0.001 | — |
| 5-Methyl-2-hexanone | 1,069 | 16.584 ± 1.676^c^ | 184.364 ± 4.557^a^ | 60.620 ± 2.807^b^ | 2.299 ± 0.451^d^ |
| 2-Heptanone | 1,463 | 5.657 ± 0.135^c^ | 27.924 ± 2.486^a^ | 1.965 ± 0.094^d^ | 8.205 ± 0.902^b^ |
| Acetoin | 1,137 | — | 103.078 ± 1.243^a^ | 12.723 ± 1.871^b^ | 7.168 ± 0.120^c^ |
| 6-Methylhept-5-en-2-one | 1,265 | — | 11.518 ± 0.414^b^ | 12.629 ± 0.936^a^ | 3.584 ± 0.551^c^ |
| 2-Nonanone | 1,386 | 10.165 ± 2.745^c^ | 46.692 ± 2.486^a^ | 36.578 ± 2.567^b^ | 2.029 ± 0.676^d^ |
| 2-Undecanone | 963 | 0.712 ± 0.003^b^ | 5.800 ± 1.203^a^ | 5.145 ± 0.281^a^ | — |

^1^ Data are expressed as the mean ± standard deviation from three replicate analyses (n = 3) of three replicate samples.

^2^ The different lowercase letters in each row indicated a significant difference between the samples (*P* < 0.05).

^3^ LrC, StC, WcC, and LhC represented samples collected at the fifth stage.

^4^ The symbol “—” indicated that the compound was not detected.

# Supplementary figures


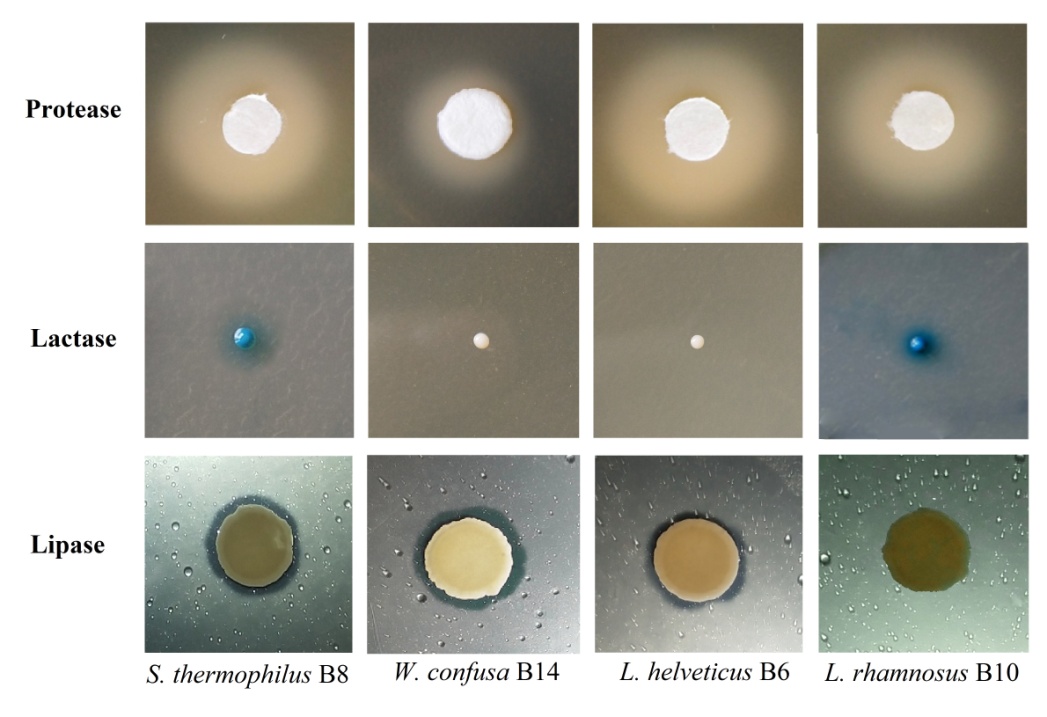


## **Figure S1.** Hydrolysis halo of selected strains


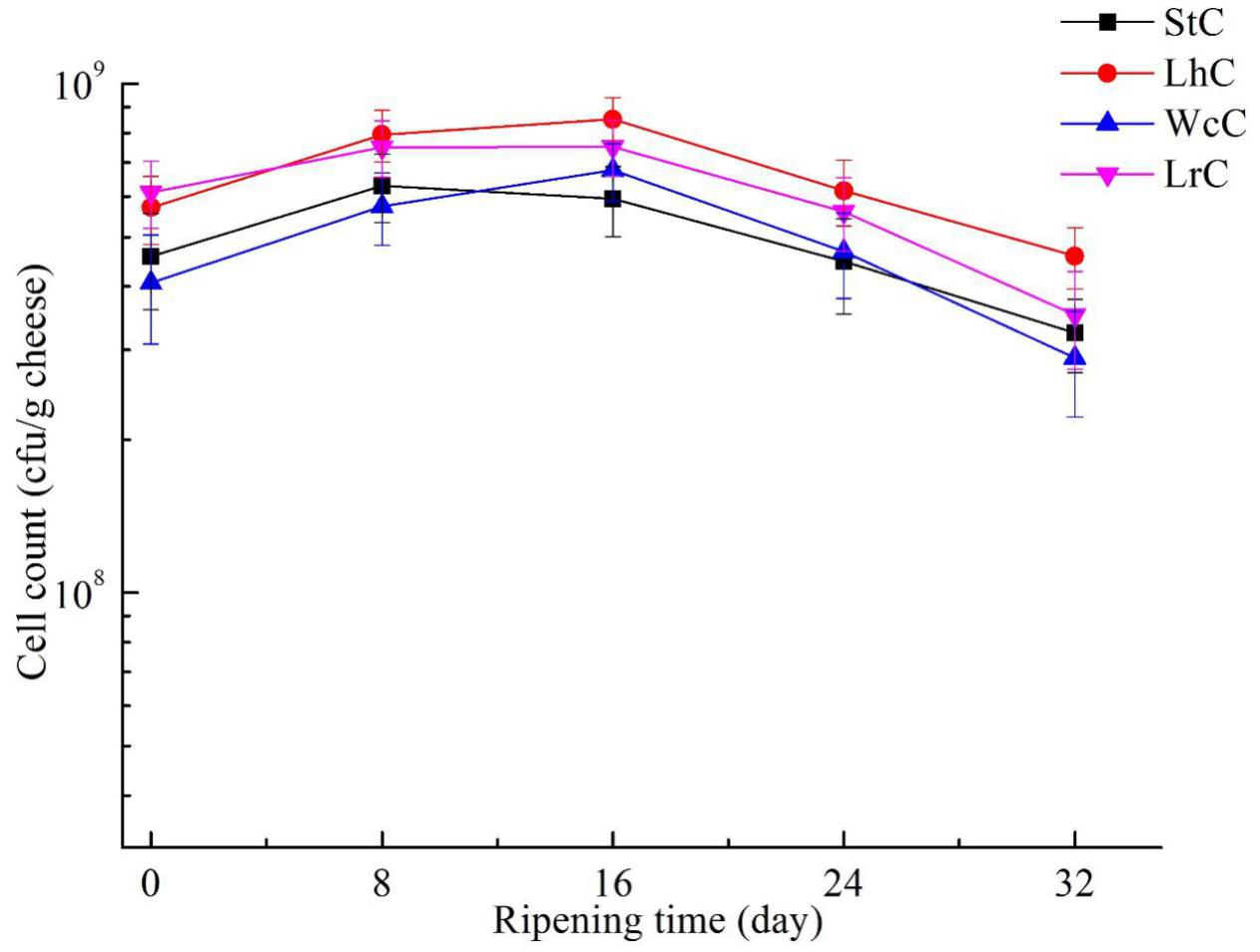


## **Figure S2.** Numbers of viable bacteria in cheeses at different fermentation periods (cfu/kg)


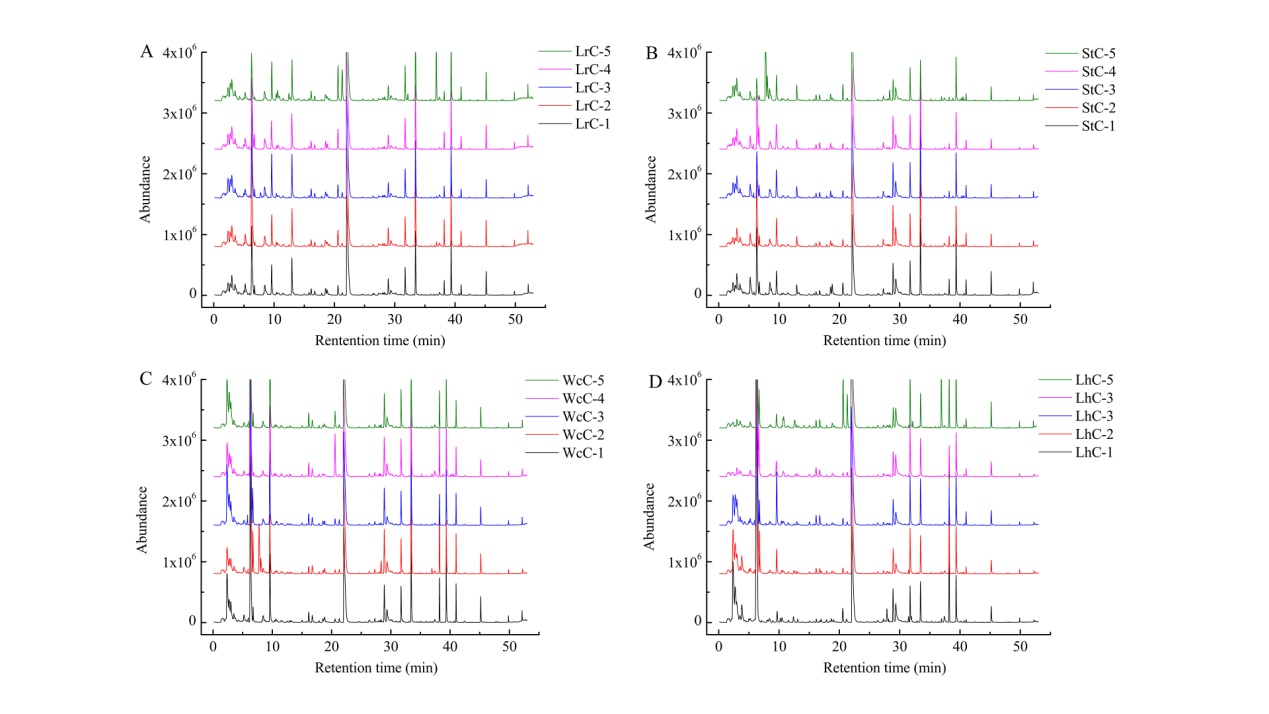


## **Figure S3.** Construction of volatile compound fingerprints for the LABs


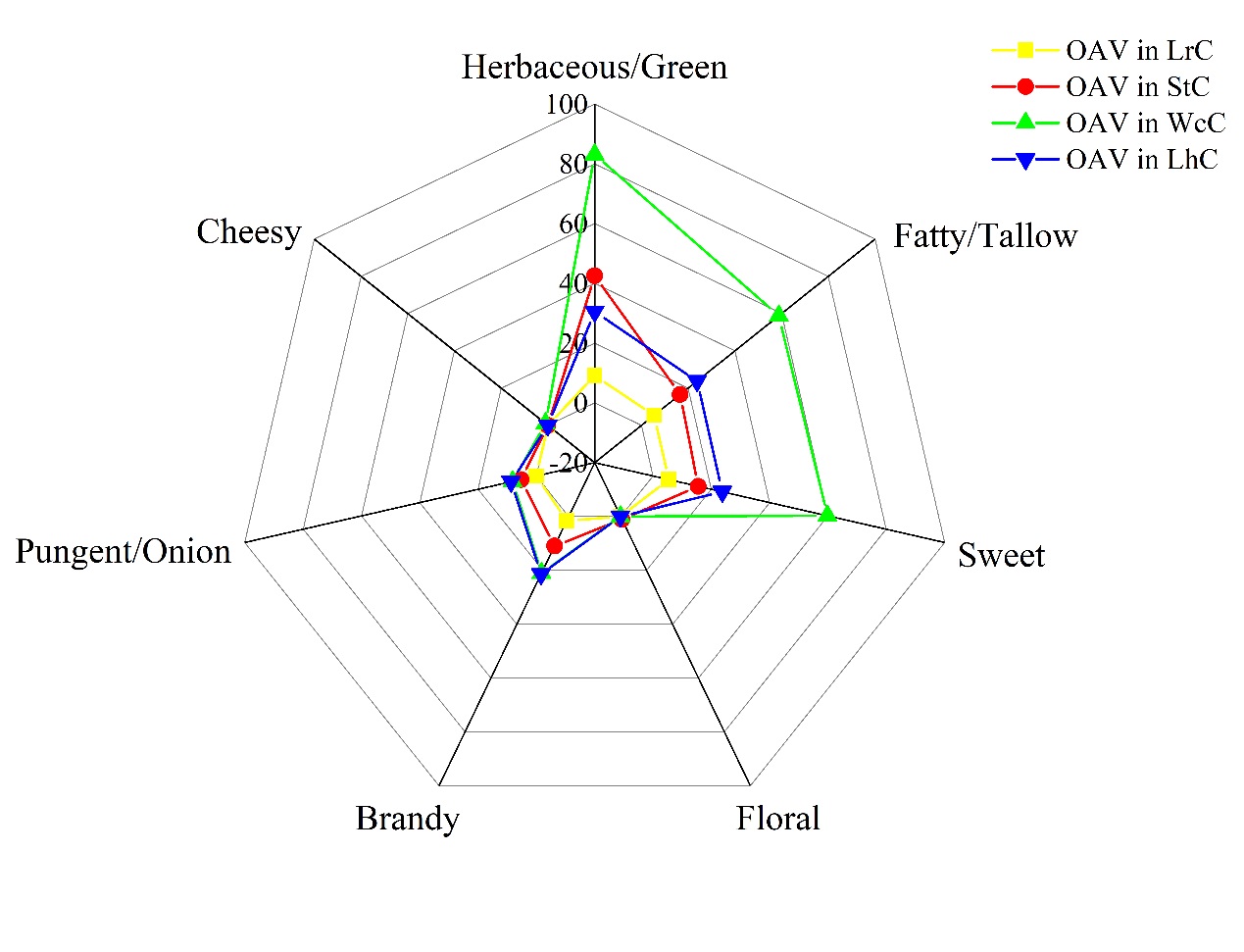


## **Figure S4.** Radar analysis of flavor compounds
